# Rosiglitazone is a superior bronchodilator compared to chloroquine and β-adrenoceptor agonists in mouse lung slices

**DOI:** 10.1186/1465-9921-15-29

**Published:** 2014-03-12

**Authors:** Chantal Donovan, Mirjam Simoons, James Esposito, Jean Ni Cheong, Meaghan FitzPatrick, Jane Elizabeth Bourke

**Affiliations:** 1 Lung Health Research Centre, Department of Pharmacology and Therapeutics, University of Melbourne, Parkville, VIC 3010, Australia

**Keywords:** Rosiglitazone, Chloroquine, β-adrenoceptor agonists, Mouse lung slices, Small airways

## Abstract

**Background:**

Current therapy for relieving bronchoconstriction may be ineffective in severe asthma, particularly in the small airways. The aim of this study was to further characterise responses to the recently identified novel bronchodilators rosiglitazone (RGZ) and chloroquine (CQ) under conditions where β-adrenoceptor agonist efficacy was limited or impaired in mouse small airways within lung slices.

**Methods:**

Relaxation to RGZ and CQ was assessed following submaximal methacholine (MCh) pre-contraction, in slices treated overnight with either RGZ, CQ or albuterol (ALB) (to induce β-adrenoceptor desensitization), and in slices treated with caffeine/ryanodine in which contraction is associated with increases in Ca^2+^ sensitivity in the absence of contractile agonist-induced Ca^2+^ oscillations. Furthermore, the effects of RGZ, CQ, ALB and isoproterenol (ISO) on the initiation and development of methacholine-induced contraction were also compared.

**Results:**

RGZ and CQ, but not ALB or ISO, elicited complete relaxation with increasing MCh pre-contraction and maintained their potency and efficacy following β-adrenoceptor desensitization. RGZ, CQ and ALB maintained efficacy following overnight incubation with RGZ or CQ. Relaxation responses to all dilators were generally maintained but delayed after caffeine/ryanodine. Pre-treatment with RGZ, but not CQ, ALB or ISO, reduced MCh potency.

**Conclusions:**

This study demonstrates the superior effectiveness of RGZ in comparison to CQ and β-adrenoceptor agonists as a dilator of mouse small airways. Further investigation of the mechanisms underlying the relatively greater efficacy of RGZ under these conditions are warranted and should be extended to include studies in human asthmatic airways.

## Introduction

There is an unmet need for novel dilators for the treatment of severe asthma, when current therapeutic agents, such as the short acting β-adrenoceptor agonist albuterol (ALB), and long acting β-adrenoceptor agonists in combination with anti-inflammatory glucocorticoids, are ineffective at completely reversing symptoms [1,2].

The peroxisome proliferator-activated receptor γ (PPARγ) agonist, rosiglitazone (RGZ) and the bitter taste receptors (TAS2R) agonists, such as chloroquine have both been recently identified as potential novel therapeutics for asthma [3-6]. PPARγ has been implicated in lung disease and the regulation of both inflammation and airway smooth muscle function [5], and its expression is upregulated in airways of asthmatic patients [7]. We have recently characterised novel PPARγ-independent dilator actions of RGZ in mouse lung slices, which are independent of K^+^-channel activation [6]. TAS2R agonists have also been shown to elicit relaxation of both large airways, potentially via large conductance Ca^2+^-activated K^+^-channels [4], and human and mouse small airways as measured in lung slices [8,9]. Critically, many subtypes of the TAS2R family are upregulated in leukocytes from severe asthmatics [10].

The limitations of current dilator agents in severe asthma may be due to their inability to overcome the increased contraction associated with airway hyperresponsiveness, or because of a loss of sensitivity due to β-adrenoceptor desensitization and/or the development of tolerance with chronic use [11,12]. The contribution of small intrapulmonary airways to this loss of dilator responsiveness is relatively understudied [13-15]. Small airways have increased sensitivity to contractile agents [16] and decreased responsiveness to β_2_-adrenoceptor agonists relative to larger airways [17]. Critical to this study, homologous desensitization has been demonstrated in small airways by culturing lung slices with high concentrations of short- or long-acting β_2_-adrenoceptor agonists [18,19]. The major aim of this study was to compare the efficacy of RGZ and CQ under conditions of both increased contraction and β-adrenoceptor desensitization.

The current study highlights the potential benefits of RGZ and CQ as novel bronchodilators for the treatment of asthma. Although less potent than ALB and isoproterenol (ISO) in mouse small airways, both RGZ and CQ elicited complete relaxation under conditions of impaired β-adrenoceptor agonist responsiveness. Both agonists maintained efficacy following overnight incubation with their respective agonist. Despite similar potency in mediating relaxation, the mechanisms of action of RGZ and CQ were distinguished by the finding that only RGZ could inhibit the initiation and development of airway contraction resulting in a marked loss of MCh potency.

## Methods

### Animals

All experimental procedures in mice were approved by the Animal Ethics Committees of the University of Melbourne (approval #1011608 and #1212485). Male Balb/C mice (6–12 weeks) were obtained from Animal Resources Centre, Western Australia, and housed in the Biomedical Sciences Animal Facility, University of Melbourne. Mice were caged at 22°C under a normal 12:12 h light:dark cycle, and given free access to a normal diet and tap water. Mice were sacrificed by an intraperitoneal injection of sodium pentobarbitone (0.40 ml, 60 mg ml^-1^, Cenvet Australia).

### Preparation of mouse lung slices

Lung slices were prepared by methods previously described [20,21]. Briefly, lungs were inflated via a tracheal cannula (20G Intima, Becton Dickinson) with warm 2% w/v ultra pure low melting point agarose (Invitrogen) in Hank’s Balanced Salt Solution supplemented with 40 mM HEPES (sHBSS) then followed by a small bolus of air. The agarose was solidified for 20 min at 4°C. A single lobe was isolated and mounted in a vibratome in cold sHBSS (VT 1000S, Leica Microsystems). 150μm thick sections were cut commencing at the lung periphery. Slices were cultured in Dulbecco’s Modified Eagle’s Medium (DMEM) supplemented with 1% penicillin-streptomycin solution, maintained at 37°C with 5% CO_2_.

### In vitro incubations for assessment of desensitization

Slices were incubated overnight with ALB (100 μM, Sigma-Aldrich Australia) to induce β-adrenoceptor desensitization, or with vehicle (water 0.1%). This concentration of ALB has been previously shown to induce β_2_-adrenoceptor desensitization in human small airways [18,19]. After incubation, airways were pre-contracted with MCh (100 nM) prior to measurement of dilator responses to ALB, ISO (Sigma-Aldrich Australia), CQ (Sigma-Aldrich Australia) or RGZ (Sapphire Bioscience Australia).

Separate slices were incubated overnight in CQ (100 μM) or water (0.1%), or RGZ (100 μM) or DMSO (0.1%). Following incubation, slices were pre-contracted with 100 nM MCh prior to measurement of dilator responses to CQ, RGZ or ALB.

### Acquisition of small airway images

Lung slice images were captured using phase contrast microscopy on an inverted microscope (Eclipse Ti-U; Nikon) with a CCD camera (model TM-62EX; Pulnix). Individual slices were placed between two cover glasses on a custom-made perfusion chamber (~100μL volume) after being covered in a fine wire mesh (Small Parts Inc.) with a small hole cut over a single airway (100–300 μm). Airways were selected for experimentation based on the presence of an intact layer of epithelial cells displaying ciliary activity and reactivity to MCh.

### Measurement of airway reactivity

Lung slices were perfused with sHBSS or varying concentrations of agonists at a constant rate using a gravity-fed perfusion system.

Some slices were treated with 20 mM caffeine and 50μM ryanodine to deplete intracellular Ca^2+^ stores and lock ryanodine receptors in an open state [20,22]. This abolishes Ca^2+^ oscillations and allows for assessment of reactivity under conditions whereby subsequent airway contraction and relaxation is due to changes in Ca^2+^ sensitivity only.

Some airways were pre-contracted with MCh prior to perfusion in the presence of K^+^ channel inhibitors alone (tetraethylammonium (TEA), glibenclamide, apamin, charybdotoxin), followed by inhibitor and chloroquine (10 μM). Inhibitor concentrations used were based on previous studies [6,23].

### Image analysis

All digital images were recorded in time lapse (0.5Hz) and analysed using image acquisition software (Video Savant; IO Industries, Inc.). A grey scale threshold was chosen to distinguish between the airway lumen and the surrounding tissue, with lumen area in each image calculated by pixel summation.

### Statistical analysis

Raw data for the airway lumen area in pixels was normalized to the initial area to correct for minor differences between airway sizes. Time course data was determined from each individual frame collected at 2 s intervals. Average constrictor and dilator responses were obtained over the last minute of each perfusion condition.

All data were expressed as mean±SEM, where each n represents a single airway per mouse. Measures of average potency (pEC_50_) and maximum responses obtained from fitted individual curves were compared by unpaired t-tests. p < 0.05 was accepted as being statistically significant. Statistical analysis was carried out using Graph Pad Prism™ (version 5.0).

## Results

### RGZ and CQ have greater efficacy than β-adrenoceptor agonists

**Figure 1:**
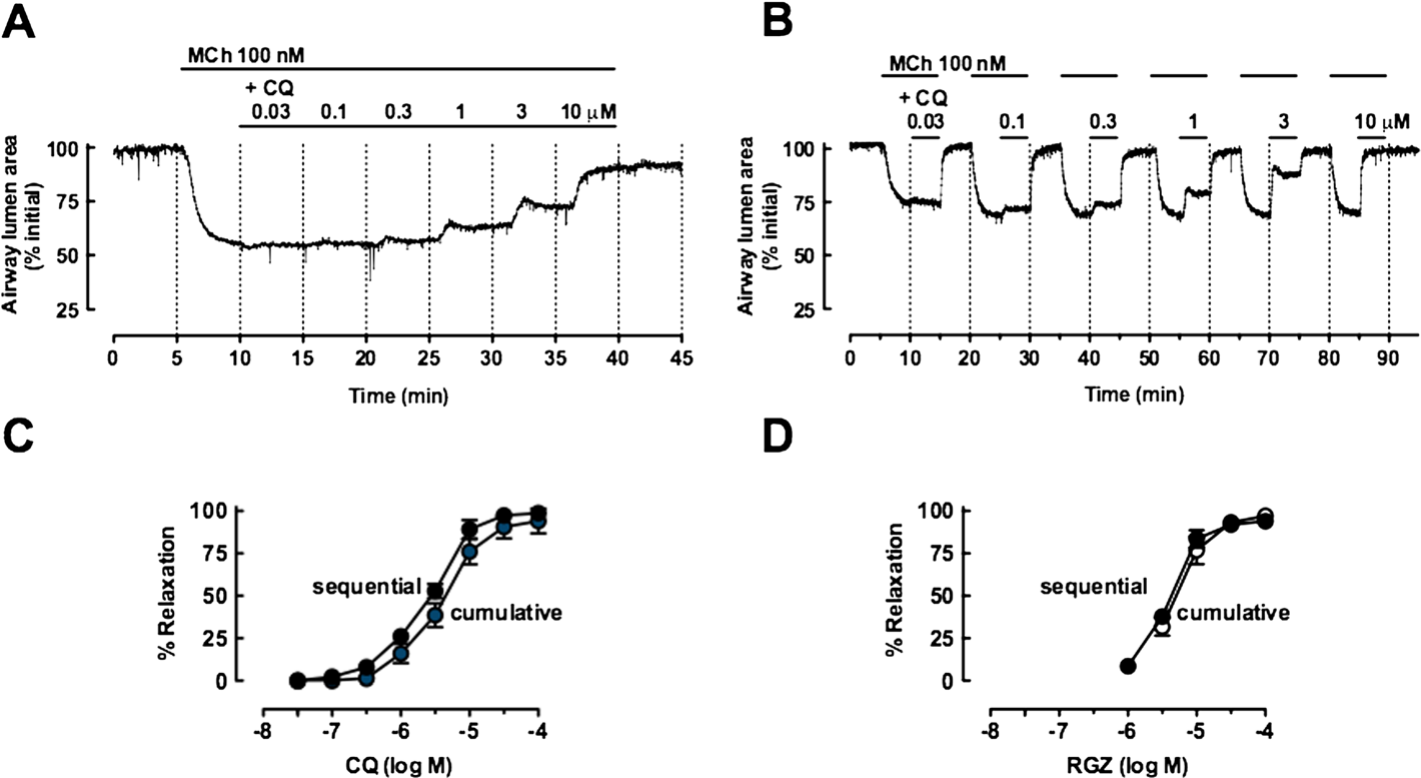
**Rosiglitazone and chloroquine elicit concentration-dependent relaxation in mouse small airways in lung slices.** Airways in lung slices were pre-contracted with 100 nM methacholine (MCh), prior to perfusion with chloroquine (CQ) or rosiglitazone (RGZ). Representative traces showing changes in airway lumen area during **A)** cumulative perfusion and **B)** sequential additions of CQ. **C) D)** Grouped data for CQ (n = 4/group) and RGZ (n = 4/group) added cumulatively or sequentially. Responses (mean ± S.E.M) represent the% relaxation of the pre-contraction to MCh, averaged over the last minute of perfusion at each dilator concentration.

We assessed the relative potency and efficacy of RGZ and CQ in mouse small airways, comparing the effects of cumulative versus sequential perfusion. A representative trace from an airway pre-contracted with a single submaximal concentration of MCh (100 nM) shows that increasing concentrations of CQ elicit complete relaxation (Figure 1A). We have previously published a trace showing that continuous perfusion with RGZ also completely reverses a submaximal contraction to MCh [6]. Similar results were obtained when repeated pre-contractions with MCh and sequential addition of increasing CQ or RGZ concentrations were separated by washout periods, demonstrating reversibility and no acute desensitization for both dilators (Figure 1B, 1C, 1D). Under the current experimental conditions, both CQ and RGZ had similar potency and efficacy (Figure 1C, 1D).

**Figure 2:**
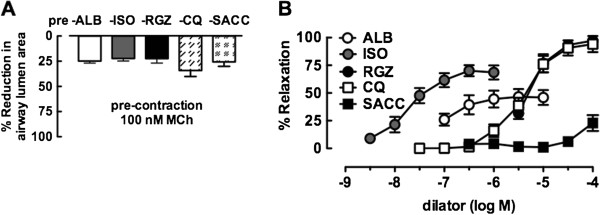
**Rosiglitazone and chloroquine, but not isoproterenol, albuterol or saccharin, elicit full relaxation of mouse small airways in lung slices.** Airways were **A)** pre-contracted with 100 nM methacholine (MCh) to achieve ~25% reduction in lumen area over 5 min prior to **B)** cumulative additions of albuterol (ALB, n = 9), isoproterenol (ISO, n = 7), rosiglitazone (RGZ, n = 4), chloroquine (CQ, n = 6) or saccharin (SACC, n = 4) at 5 min intervals. Responses (mean ± S.E.M) represent **A)** the reduction in lumen area to MCh and **B)** the% relaxation of this pre-contraction averaged over the last minute of each perfusion.

In airways pre-contracted to a similar extent (Figure 2A), cumulative concentration-response curves to RGZ and CQ were compared with those for the β-adrenoceptor agonists ALB and ISO (Figure 2B). RGZ and CQ had similar potency and caused complete relaxation at the maximal concentrations tested (% relaxation to 100 μM CQ: 105 ± 3%, 100 μM RGZ 107 ± 9%). Although ALB and ISO were relatively more potent (pEC_50_; ISO: 7.9 ± 0.2), neither of the β-adrenoceptor agonists fully reversed the MCh-mediated contraction (Figure 2B). Saccharin, an agonist of the bitter taste receptors, only produced partial relaxation (22.9 ± 7.2%) at very high μM concentrations.

### Relaxation to RGZ and CQ, but not β-adrenoceptor agonists, is maintained with increased levels of MCh pre-contraction

**Figure 3:**
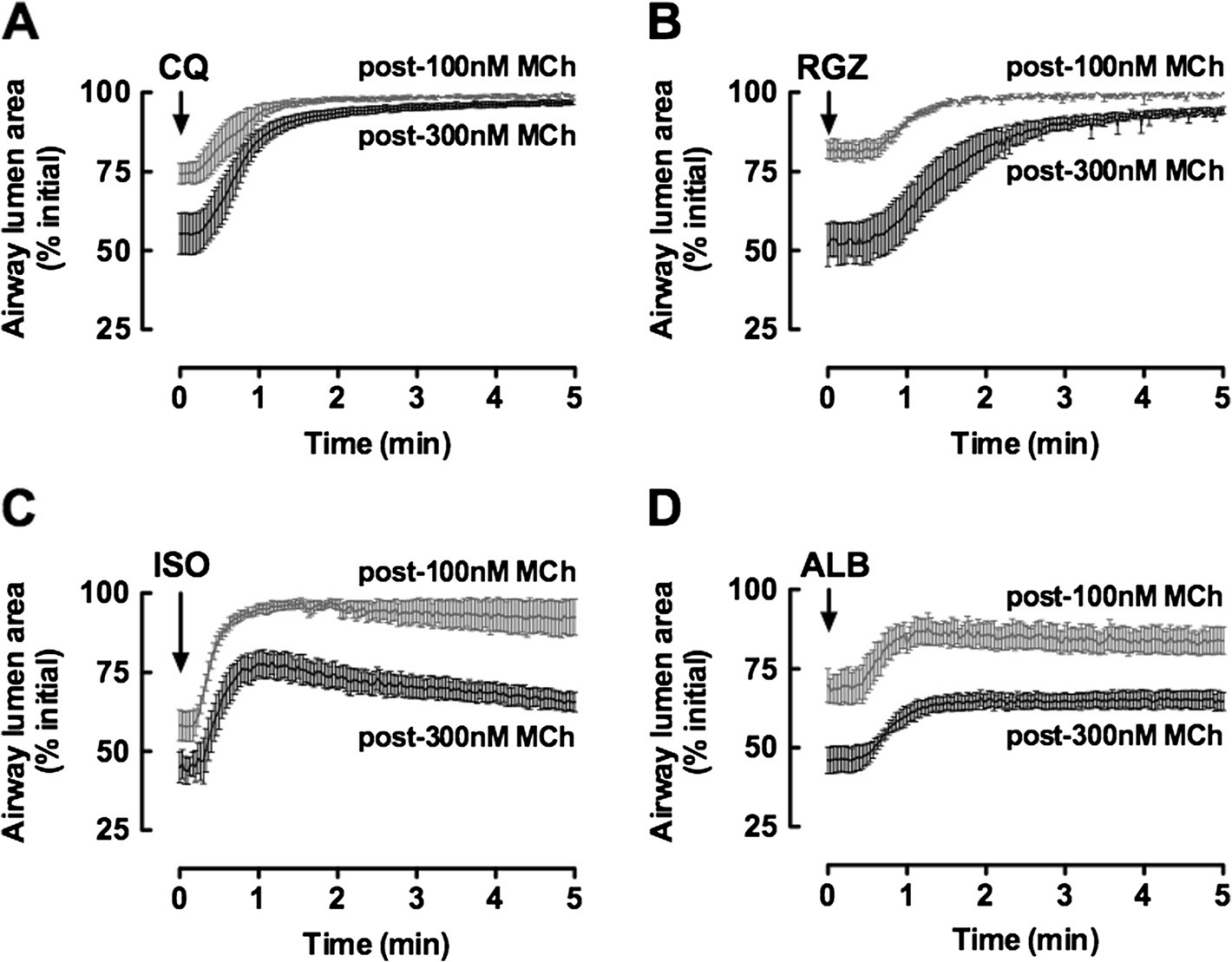
**Rosiglitazone and chloroquine and, but not isoproterenol and albuterol, can overcome increased contraction to methacholine.** Airways in lung slices were pre-contracted with 100nM methacholine (MCh) or 300nM MCh, prior to 5 min perfusion with **A)** chloroquine (CQ, 100 μM, n = 4, 8), **B)** rosiglitazone (RGZ, 100 μM, n = 3, 4), **C)** isoproterenol (ISO, 10 μM, n = 3, 4) or **D)** albuterol (ALB, 10 μM, n = 4, 4). Responses (mean ± S.E.M) represent the average airway lumen area (normalized to the initial area), measured at 2 sec intervals over 5 min.

Maximally effective concentrations of each bronchodilator (established in Figure 2) were used to assess their relative abilities to overcome a greater level of pre-contraction, using airways pre-contracted with either 100 nM or 300 nM MCh. Complete relaxation to both CQ and RGZ was maintained (Figure 3A, 3B), but the partial relaxation in response to both ISO and ALB was reduced against 300 nM MCh (Figure 3C, 3D). The time of onset of all bronchodilator responses was unchanged at the differing levels of MCh-induced contraction.

### Pre-treatment with RGZ, but not CQ or β-adrenoceptor agonists, reduces MCh potency

**Figure 4:**
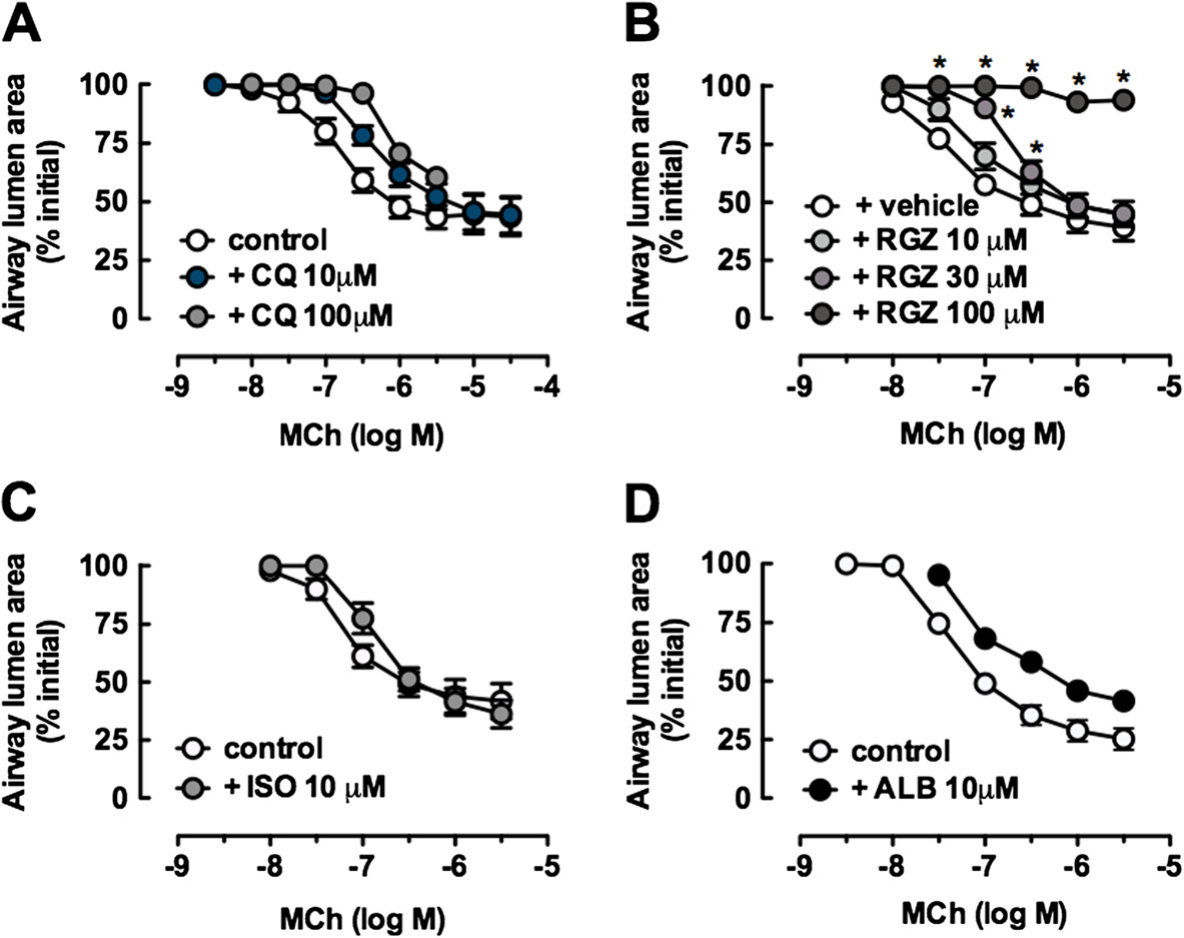
**Rosiglitazone, but not chloroquine, isoproterenol or albuterol, inhibits the initiation and development of methacholine-induced contraction.** Lung slices were perfused with increasing concentrations of methacholine (MCh) in the presence of **A)** chloroquine (CQ) (control: n = 8, 10 μM: n = 8, 100 μM: n = 3), **B)** rosiglitazone (RGZ) (vehicle: n = 4, 10 μM: n = 3, 30 μM: n = 4, 100 μM: n = 4), **C)** isoproterenol (ISO) (control: n = 4, 10 μM: n = 4) or **D)** albuterol (ALB) (control n = 4, 10 μM, n = 4). Responses (mean ± S.E.M) represent the airway lumen area (normalized to the initial area), averaged over the last minute of perfusion at each MCh concentration. *p < 0.05, 2 way ANOVA, Bonferroni *post-hoc*.

To extend these observations, we then assessed the ability of these dilators to inhibit the initiation and development of MCh-induced contractions, using concentrations of RGZ and CQ shown to relax pre-contracted airways. Following perfusion in the presence of CQ, RGZ, ALB or ISO, airways were exposed to increasing concentrations of MCh (Figure 4). There was a decrease in MCh potency in the presence of 10 μM CQ (MCh pEC_50_: vehicle 6.9 ± 0.1; +10 μM CQ 6.3 ± 0.1, p < 0.05), but this was not further reduced with 100 μM CQ (Figure 4A). The presence of both 30 μM and 100 μM RGZ significantly decreased the potency of MCh (MCh pEC_50_: vehicle 7.4 ± 0.1, +30μM RGZ 6.6 ± 0.0, p < 0.05) (Figure 4B). 100 μM RGZ also decreased the maximum contraction to MCh (Figure 4B). Neither ISO nor ALB reduced the potency of MCh or the maximum reduction in airway lumen area (MCh pEC_50_: control 7.3 ± 0.05, +10 μM ALB 7.4 ± 0.17, NS) (Figure 4C, 4D).

### RGZ and CQ, but not β-adrenoceptor agonists, are resistant to desensitization

**Figure 5:**
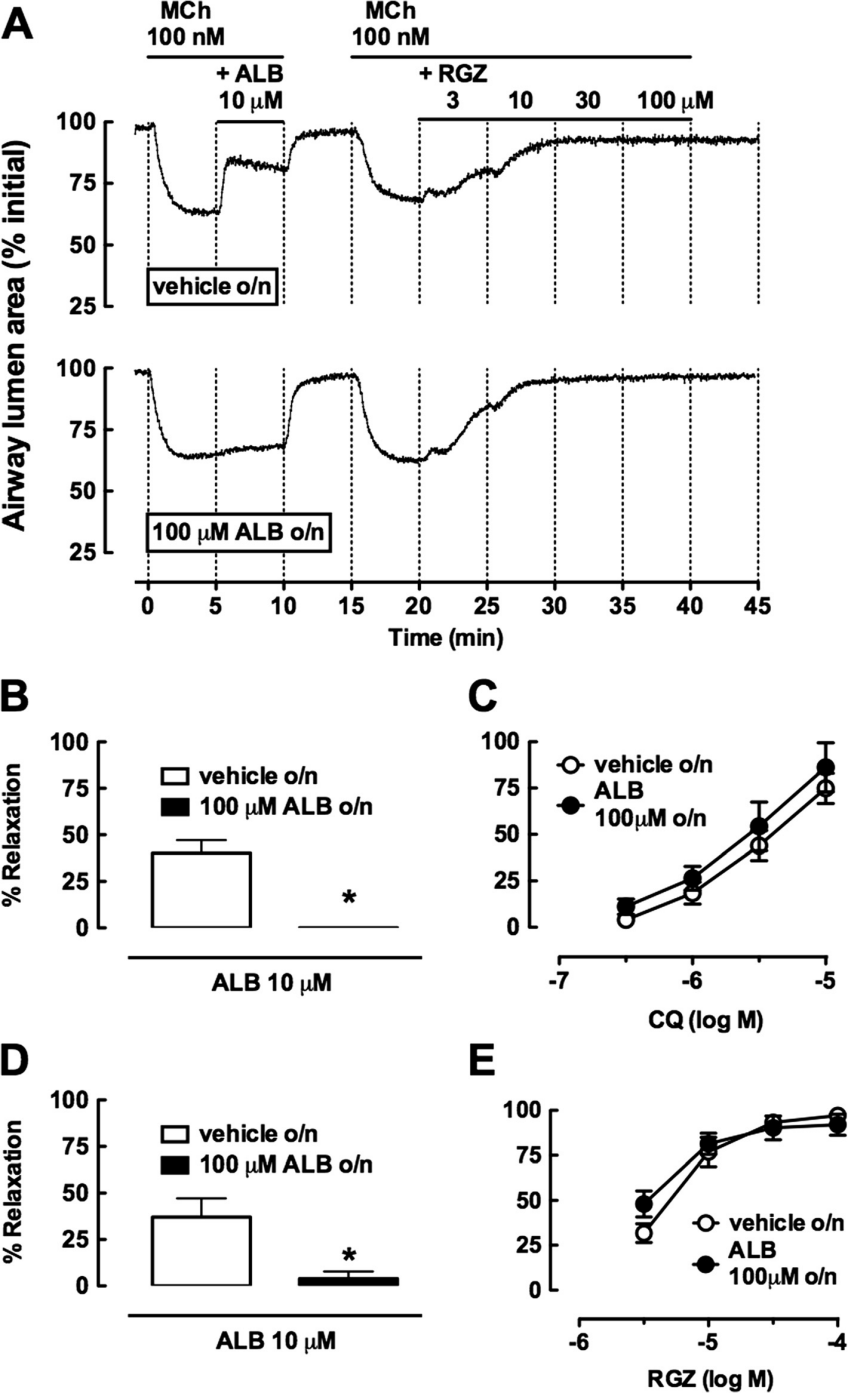
**Rosiglitazone and chloroquine maintain potency and efficacy despite β-adrenoceptor desensitization.** Slices were incubated overnight with vehicle (0.1% water) or albuterol (ALB, 100 μM). Airways were then pre-contracted with 100 nM methacholine (MCh) prior to addition of 10 μM ALB and concentration response curves to rosiglitazone (RGZ) or chloroquine (CQ). **A)** Representative traces illustrating RGZ concentration-response curves following overnight incubation with vehicle or ALB. Relaxation responses to **B) C)** 10 μM ALB and CQ (n = 4/group) and **D) E)** 10 μM ALB and RGZ (n = 4/group) following overnight incubation with vehicle or 100 μM ALB. *p < 0.05, unpaired *t*-test.

To compare whether dilator responses to RGZ, like CQ, were resistant to heterologous desensitization, lung slices were incubated overnight with ALB (100 μM). Under these conditions, β-adrenoceptor-mediated relaxation to ALB was completely abolished (Figure 5A, 5B, 5D), and relaxation to ISO (10 μM) was also significantly reduced (maximum relaxation after vehicle o/n: 54.0 ± 7.0%, after ALB o/n: 11.7 ± 6.7%, p < 0.05). Despite β-adrenoceptor desensitization, concentration-dependent relaxation to both CQ and RGZ was maintained with similar potency and maximum (Figure 5A, 5C, 5E).

**Figure 6:**
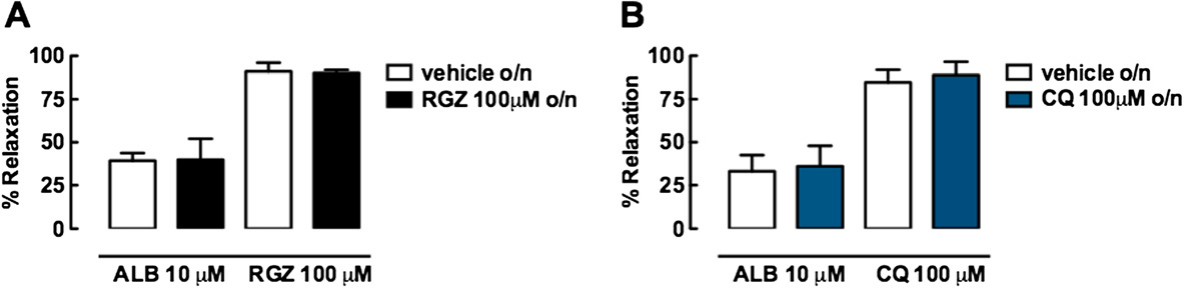
**Rosiglitazone and chloroquine maintain efficacy following overnight incubation with CQ or RGZ.** Slices were incubated overnight with vehicle (0.1% water for chloroquine (CQ), 0.1% DMSO for rosiglitazone (RGZ)) or CQ (100 μM) or RGZ (100 μM). Airways were pre-contracted with 100 nM methacholine (MCh) prior to addition of albuterol (ALB, 10 μM) or CQ (100 μM) or RGZ (100 μM). **A)** Relaxation response to ALB and RGZ following overnight incubation with RGZ (n = 3/group). **B)** Relaxation response to ALB and CQ following CQ incubation overnight (n = 3/group).

To determine whether relaxation to RGZ or CQ was impaired following overnight incubation with the same dilator, slices were incubated with 100 μM of either agonist or appropriate vehicles. Both RGZ and CQ elicited similar relaxation following these overnight incubations (Figure 6A, 6B). Relaxation to ALB (10 μM) was also maintained irrespective of the incubation conditions (Figure 6A, 6B).

### All dilators elicit delayed relaxation in caffeine/Ryanodine treated slices

**Figure 7:**
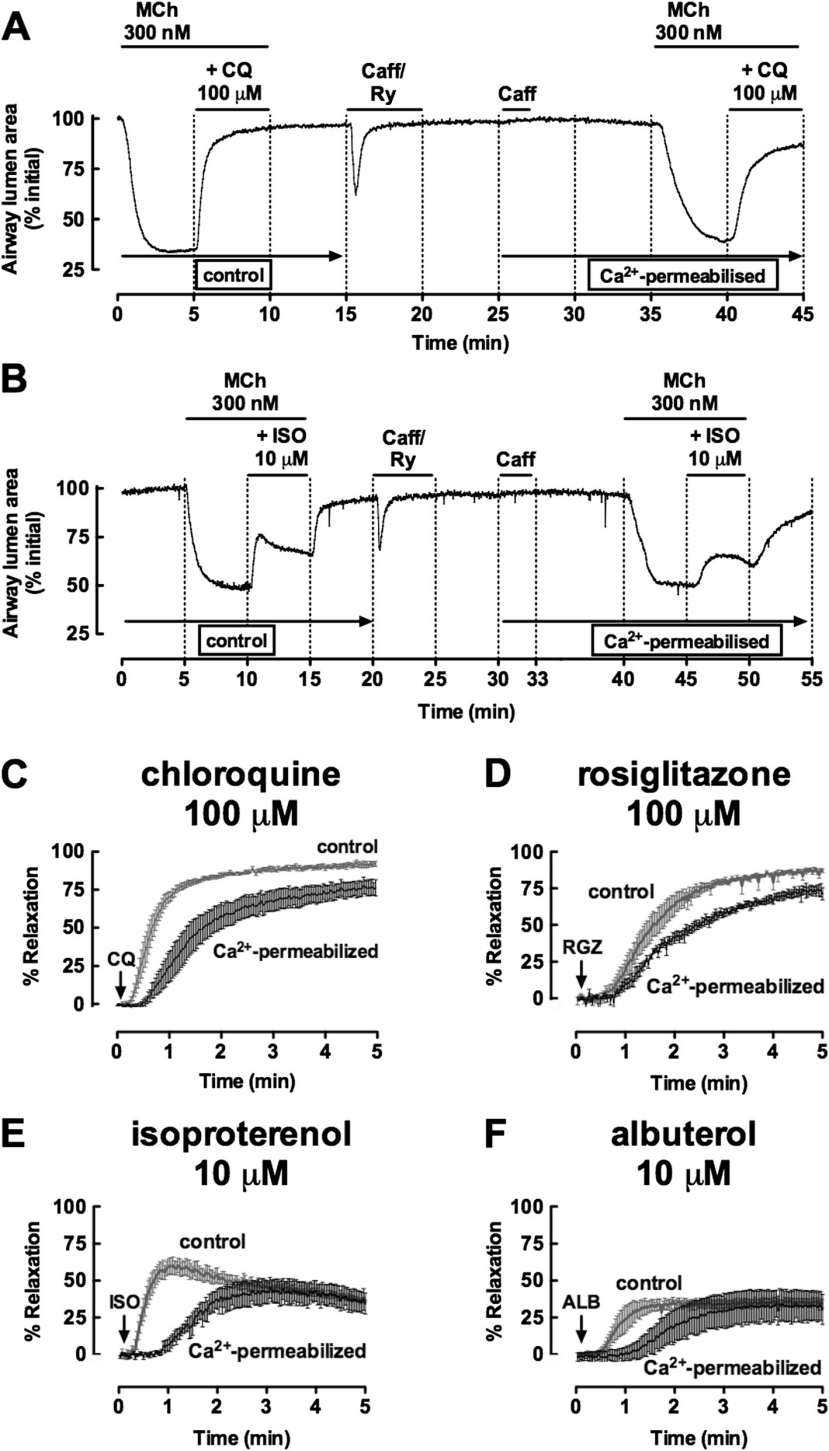
**Rosiglitazone and chloroquine and oppose airway contraction due to calcium sensitivity.** Small airways in lung slices were pre-contracted with 300 nM methacholine (MCh), prior to perfusion with dilators for 5 min. Representative traces show changes in airway lumen area for relaxation to **A)** chloroquine (CQ) and **B)** isoproterenol (ISO) before and after treatment with caffeine/ryanodine. Grouped data for **C)** CQ (100 μM), **D)** rosiglitazone (RGZ, 100 μM), **E)** ISO (10 μM) and **F)** albuterol (ALB, 10 μM) (n = 4/group) show pre- and post-caffeine/ryanodine responses within the same slice. Responses (mean ± S.E.M) represent the% relaxation of the MCh pre-contraction measured at 2 sec intervals over 5 min.

To compare the capacity of RGZ and CQ to overcome small airway contraction due to Ca^2+^-sensitivity alone, reactivity was assessed after treatment with caffeine (20 mM)/ryanodine (50 μM) to induce a transient contraction and to lock ryanodine receptors in an open state to clamping intracellular Ca^2+^ levels. Subsequent exposure to caffeine did not cause contraction, confirming that subsequent responses could be attributed to altered calcium sensitivity alone (Figure 7A, 7B).

Relaxation responses to a single concentration of CQ or RGZ were relatively slower after caffeine/ryanodine treatment such that a plateau response was not achieved within 5 min (Figure 7A, 7C, 7D). The average relaxation response measured over the final minute of the perfusion was slightly reduced for both dilators (RGZ control: 86.1 ± 1.1%, post caffeine/ryanodine: 70.4 ± 2.9%, p < 0.05, Figure 7D). However, relaxation to CQ and RGZ (Figure 7C, 7D) was still greater compared with either ISO or ALB (Figure 7B, 7E, 7F).

Under control conditions, both β-adrenoceptor agonists elicited rapid but only partial relaxation. After caffeine/ryanodine treatment, the peak responses associated with this rapid relaxation were lost but despite the delayed onset, both agonists elicited gradual relaxation of similar magnitude to the control responses by the end of 5 min perfusion (Figure 7B, 7E, 7F).

Time-control experiments established that there was no difference between the first and second exposure to any agonist tested, indicating that the impairment of relaxation to all agonists tested was due to caffeine/ryanodine treatment and not acute desensitization caused by the initial 5 min exposure period (data not shown).

### CQ-mediated relaxation is inhibited by TEA but not by selective K^+^-channel inhibitors

Given conflicting reports in the recent literature of the mechanism underlying CQ-mediated relaxation, responses to CQ in small airways were compared in the absence or presence of various K^+^-channel inhibitors (Table 1). The non-selective K^+^-channel inhibitor TEA alone partially reversed the reduction in lumen area in the presence of MCh, and subsequent relaxation in response to perfusion with CQ in the continued presence of TEA was impaired. In contrast, TEA had previously been shown to have no effect on relaxation to RGZ in mouse lung slices [6].

**Table 1:** The effect of potassium channel inhibitors on chloroquine-mediated relaxation of mouse small airways

	**n**	**% Relaxation**	
		**Inhibitor alone**	**Inhibitor + CQ**
Vehicle	8	1.2 ± 2.1	88.1 ± 4.9
TEA (1 mM)	4	36.3 ± 7.8***	39.8 ± 3.2^^^
Glibenclamide (1 μM)	4	0.6 ± 2.4	89.3 ± 2.9
Apamin (500 nM)	4	0.1 ± 2.5	92.8 ± 3.7
Charybdotoxin (10 nM)	4	5.2 ± 2.1	83.4 ± 11.1

Airways were pre-contracted with 100nM MCh prior to perfusion for 5 min in the presence of inhibitor alone, followed by inhibitor and chloroquine (10 μM). Responses (mean ± S.E.M) represent the% relaxation of the pre-contraction to MCh, averaged over the last minute of perfusion. ***p < 0.0001 *cf* vehicle. ^^^p < 0.0001 *cf* CQ alone.

Assessment of the effects of more selective inhibitors of K^+^-channels on CQ-mediated relaxation was undertaken. Neither the ATP-sensitive K^+^-channel inhibitor, glibenclamide, the small conductance Ca^2+^-activated K^+^-channel inhibitor apamin, nor the high conductance Ca^2+^-activated K^+^-channel inhibitor, charybdotoxin alone altered the contractile response to MCh, and full relaxation to CQ was maintained.

## Discussion

In the present study, we have extended previous studies exploring responses to RGZ and CQ in small airways in mouse lung slices [6,9], to further characterize their relative therapeutic potential to oppose airway constriction in asthma. We confirmed that these dilators were less potent than β-adrenoceptor agonists [4,6], but unlike ALB and ISO, both RGZ and CQ were able to elicit complete relaxation at μM concentrations, even with increasing levels of MCh-induced pre-contraction. Critically, we have now demonstrated that both RGZ and CQ maintain their efficacy under conditions of β-adrenoceptor desensitization, with neither dilator causing homologous desensitization or reducing the sensitivity of mouse small airways to β-adrenoceptor agonists. However, only RGZ was able to inhibit the initiation and development of maximal MCh-induced contraction, suggesting additional potential benefit over CQ as a novel dilator for the treatment of asthma.

Current therapeutic approaches to relieve asthma symptoms with inhaled β-adrenoceptor agonists are generally effective, however, a significant proportion of patients have poorly controlled asthma [2,24]. It is for this group of patients that alternative or supplementary dilator therapies may provide benefit. We have assessed two distinct novel bronchodilators in mouse small airways. RGZ, an agonist for PPARγ, has been shown to elicit acute relaxation of pre-contracted mouse trachea and small airways [6,25]. CQ, a bitter taste receptor agonist, has been shown to activate TAS2R present on airway smooth muscle and cause bronchodilation [4].

There has been increased recent interest in targeting the small airways, a major site of airway obstruction in difficult-to-treat patients. Both RGZ and CQ were able to elicit relaxation at lower potency but higher efficacy than the β-adrenoceptor agonists ISO and ALB in mouse small airways. The relative potency of CQ compared with ISO in small airways (>300-fold lower) is smaller than the 10,000-fold difference reported previously in mouse trachea [4,26] but remains consistent with the low affinity of TAS2Rs compared to other GPCRs, such as the β-adrenoceptor. We also found an additional TAS2R agonist, SACC to be >100 fold less potent than CQ in the small airways. This is in agreement with minimal response to SACC previously reported in human and mouse airways [9,27].

Since increasing levels of contraction are known to decrease the efficacy of various dilators [6,28], we assessed all four dilators at two levels of MCh-induced contraction. While the β-adrenoceptor agonists were susceptible to functional antagonism, both CQ and RGZ were able to overcome a higher pre-contraction. This is consistent with previous findings that CQ (100 μM) and RGZ (100 μM) could elicit complete relaxation at up to 300 and 100 times higher concentrations of MCh respectively [6,9].

To extend this observation, we also examined the ability of each dilator to prevent the initiation and development of MCh-induced contraction. While pre-treatment with ISO and ALB had no significant effect on the MCh concentration- response curve, both CQ and RGZ were able to inhibit the reduction in airway area at low MCh concentrations. However only RGZ, but not CQ, reduced both MCh potency and the maximum contraction. This finding in the mouse contrasts with guinea pig trachea where pre-incubation with CQ, at a supramaximal concentration of 300 mM, inhibited the development airway contraction to several contractile mediators, including carbachol [29].

Desensitization of GPCRs may limit the therapeutic efficacy of agonists by rapidly attenuating the early signaling that leads to the cellular response. A reduction of bronchodilator efficacy due to β-adrenoceptor desensitization is a well-recognized limitation of β-adrenoceptor agonists [30,31]. Given this background, we assessed whether sequentially or cumulative administration of CQ or RGZ would influence their efficacy in mouse small airways. Our experimental traces suggested that increasing concentrations of CQ induced rapid initial peak dilator response that could not be maintained. However, its similar potency and efficacy in cumulative concentration-response curves suggests that the relaxation to higher concentrations of CQ was not limited by acute receptor desensitization. Similar findings with RGZ illustrate that both dilators are reversible even at the highest concentrations tested.

Recent reports have suggested that CQ promotes homologous desensitization of human airway smooth muscle TAS2Rs occurring as early as 5 min after exposure to quinine and becoming progressively greater with longer incubation times [32]. Given that we did not see any acute desensitization to either CQ or RGZ, we assessed whether overnight incubation at high concentrations would induce either homologous desensitization or heterologous desensitization to ALB. We found no evidence of impaired relaxation to either dilator and are the first to demonstrate no heterologous desensitization of the β-adrenoceptor, as seen by the maintained response to ALB.

These findings may indicate that the TAS2R subtypes present on mouse airway smooth muscle may not be prone to desensitization via the G-protein-coupled receptor kinase (GRK) as previously suggested in human airway smooth muscle cells [32,33]. It does provide crucial evidence that RGZ and CQ do not cross-desensitize the β-adrenoceptor and that future combination therapy with these dilators and β-adrenoceptor agonists in current use is viable.

Homologous β-adrenoceptor desensitization was also established in lung slices following persistent exposure to ALB. Treatment with ALB almost completely abolished subsequent ALB-mediated relaxation in mouse small airways. The response to the β_1_/β_2_-adrenoceptor agonist ISO was only partially inhibited, consistent with the contribution of β_1_-adrenoceptors to mouse airway relaxation [34]. This is the first study to show that RGZ and CQ can maintain potency and complete small airway relaxation following β-adrenoceptor desensitization in mouse lung slices. The finding is consistent with previous results obtained with CQ in mouse trachea and human precision cut lung slices following 18 h incubation with ISO or the long acting β-adrenoceptor agonist salmeterol respectively [8]. The ability of both RGZ and CQ to cause relaxation under conditions of β-adrenoceptor desensitization provides further evidence that these dilators are working via a different mechanism to β-adrenoceptor agonists.

We explored another potential mechanism downstream of the β-adrenoceptor itself that could contribute to RGZ- and CQ-induced relaxation. It has been established that relaxation to both short-acting β-adrenoceptor agonists and RGZ is associated with inhibition of MCh-induced increases in both Ca^2+^-oscillations and Ca^2+^-sensitivity [6,35]. We compared the effects of CQ with RGZ, ISO and ALB after caffeine/ryanodine treatment to assess whether relaxation to CQ could also be maintained when the contraction to MCh was due to Ca^2+^-sensitivity alone.

CQ and RGZ appeared to be slightly less effective at reversing MCh pre-contraction than under control conditions, while there was a notable loss of the rapid phase of the β-adrenoceptor-mediated relaxation. The slower rate of relaxation to CQ and RGZ after caffeine/ryanodine treatment suggests that a longer perfusion period with either dilator might be necessary to determine whether it can fully overcome the increase in Ca^2+^-sensitivity in response to MCh. However these novel dilators maintained their greater efficacy than ISO or ALB under these conditions.

Consideration of the potential contribution of the regulation of Ca^2+^-sensitivity by CQ was important, as this had not been addressed in the initial study describing dilator responses to bitter-taste compounds [4]. A paper published during the preparation of this manuscript confirms that bitter-taste compounds such as CQ reduce Ca^2+^ sensitivity in mouse small airways, and also demonstrates that CQ can inhibit agonist-induced Ca^2+^ oscillations when reversing bronchoconstriction [9]. As such, these findings have yet to differentiate between the mechanisms of action of CQ and RGZ, which also inhibits Ca^2+^signaling and sensitivity [6].

There are conflicting reports of the contribution of K^+^-channel activation to CQ-mediated relaxation. Although BK_Ca_-channels had initially been implicated in the dilator response to TAS2R agonists in mouse trachea [4], a subsequent report did not replicate this finding, with relaxation to CQ maintained in the presence of iberiotoxin or two other BK_Ca_-channel-blockers, and currents through BK_Ca_-channels unaltered by CQ [36].

Our results obtained in mouse small airways showed that the nonselective K^+^-channel inhibitor TEA alone caused partial reversal of the MCh pre-contraction. This may be a consequence of hyperpolarization of airway smooth muscle by TEA, disturbing the balance of in- and outwardly rectifying K^+^-currents regulating the resting membrane potential [37]. Following this partial relaxation to TEA, the subsequent relaxation by CQ in the presence of TEA was almost completely abolished, suggesting that this TAS2R agonist was at least partially dependent on K^+^-channel activation for its relaxant effect in mouse small airways.

Despite this finding, we were unable to implicate a specific K^+^-channel in CQ-mediated relaxation in mouse small airways. Neither the ATP-sensitive K^+^-channel inhibitor glibenclamide, the small conductance Ca^2+^-activated K^+^-channel inhibitor apamin nor the large conductance and voltage-gated K^+^-channel inhibitor charybdotoxin affected the MCh-induced stable contraction or relaxation to CQ. Additional mechanisms mediating CQ-induced airway relaxation are likely, with further studies required to assess which K^+^-channels may be involved.

It is also possible that different mechanisms are implicated depending on the preparation in which the actions of CQ are assessed. Studies in isolated mouse airway smooth muscle cells and isolated trachea showed that CQ inhibited MCh-induced Ca^2+^ signaling and reversed contraction by blocking voltage-gated Ca^2+^ channels (VGCC) via Gβγ or Gα_i_ signaling [38]. However, in mouse small airways in lung slices, VGCC do not play a major role in MCh-mediated contraction [39]. In this setting, bronchodilation with bitter taste compounds was unaffected by blockade of Gβγ or Gα_i_ signaling, suggesting that it could be directed by Gα-gustducin or other G-proteins known to associate with TAS2R [9].

The results with CQ are in contrast to RGZ, as relaxation to RGZ has been shown to be maintained in the presence of TEA [6]. The relative potential of these dilators to provide relief for patients with difficult-to-treat asthma may be informed by the previous clinical evidence that K^+^-channels openers have had limited therapeutic benefit in asthma treatment [40,41]. However, most of the bronchodilators that activate K^+^-channels are also known to exert other effects on airway smooth muscle, such as decreasing Ca^2+^ sensitivity [42]. Nevertheless, the finding that RGZ-mediated dilation is resistant to TEA but can still overcome contraction due to Ca^2+^-sensitivity alone [6], provides additional pre-clinical evidence to support its potential benefit in asthma treatment.

## Conclusion

Both RGZ and CQ, but not β-adrenoceptor agonists, were able to elicit complete relaxation with similar potency in mouse small airways, which was maintained under conditions of homologous β_2_-adrenoceptor desensitization. Although both RGZ and CQ could overcome contraction due to calcium sensitivity alone, only RGZ inhibited the initiation and development of a maximal contraction to MCh. Further elucidation of their mechanisms of action is warranted, to support their clinical assessment as novel bronchodilators targeting small airways in patients with poorly controlled asthma. Further investigation of the mechanisms underlying the superior dilator responsiveness to RGZ in comparison to CQ and β-adrenoceptor agonists under these conditions are warranted and should be extended to human asthmatic airways.

## Abbreviations

CQ: Chloroquine; ISO: Isoproterenol; MCh: Methacholine; PPAR: Peroxisome-proliferator-activated receptor; RGZ: Rosiglitazone; ALB: Albuterol.

## Competing interests

The authors declare that they have no competing interests.

## Authors’ contributions

CD, MS, JE, JNC, MF conducted all experiments. CD, MS, JE, JNC, MF and JEB analyzed data and conceptualized project. CD, MS, JEB wrote the paper. CD, MS, JE, JNC, MF and JEB approved the submission.
